# Impact of chemotherapy on perceptions related to food intake in women with breast cancer: A prospective study

**DOI:** 10.1371/journal.pone.0187573

**Published:** 2017-11-30

**Authors:** Eduarda da Costa Marinho, Isis Danyelle Dias Custódio, Isabela Borges Ferreira, Cibele Aparecida Crispim, Carlos Eduardo Paiva, Yara Cristina de Paiva Maia

**Affiliations:** 1 Graduate Program in Health Sciences, Federal University of Uberlandia, Uberlandia, Minas Gerais, Brazil; 2 Nutrition Course, Medical Faculty, Federal University of Uberlandia, Uberlandia, Minas Gerais, Brazil; 3 Department of Clinical Oncology, Graduate Program in Oncology, Palliative Care and Quality of Life Research Group (GPQual), Pio XII Foundation-Barretos Cancer Hospital, Barretos, Sao Paulo, Brazil; University of South Alabama Mitchell Cancer Institute, UNITED STATES

## Abstract

Breast cancer (BC) treatment includes mostly chemotherapy (CT), which can cause side effects like nausea, taste changes, early satiety, slow gastric emptying and xerostomia. In this way, the individual’s relationship with food may change during the treatment. The aim of this study was to evaluate the impact of chemotherapy on perceptions related to food intake of women with BC. Fifty-five women with BC were followed, and data were collected at three periods during first-line CT: beginning (T0), intermediate (T1) and end (T2). A visual analogue scale (VAS) (0 to 10 cm) for hunger, appetite for various food categories and meal enjoyment was investigated. The frequency and intensity of side effects were evaluated using a 4 cm scale. The results showed a higher prevalence of taste changes in T1 (p = 0.044) and more nausea in T1 and T2 (p = 0.018). Furthermore, the intensity of nausea was higher in T2 (p = 0.01) than in the other periods. We observed moderate hunger in T0, T1 and T2 (p = 0.113), but the overall appetite increased between T0 and T2 (p = 0.003). Meal enjoyment was reduced from T0 to T1and returned back to the initial value in T2 (p = 0.021). The appetite for salty (p = 0.004) and spicy (p = 0.03) foods was increased in T1. There was an increase of body weight (p = 0.008), body mass index (BMI) (p = 0.009) and waist circumference (WC) (p = 0.03) during CT. CT changes food hedonism, increasing the overall appetite and the appetite for salty and spicy foods. Moreover, we observed the negative impact of CT on meal enjoyment and an increase in side effects and anthropometric parameters.

## Introduction

Breast cancer (BC) treatment includes chemotherapy (CT), encompassing a group of substances that act on various steps of cellular metabolism [1]. The biological aggressiveness of CT harms cells in the digestive tract causing early satiety, slower gastric emptying, xerostomia [2], nausea, vomiting and taste changes [3]. In consequence of the symptoms caused by CT, the individual’s relationship with food may change during treatment; tasty preparations before CT become unattractive or cause discomfort [4]. The reduced meal appreciation during CT has negative physical, emotional and social consequences [5].

In spite of changes in perception being related to the reduction of food intake, many patients gain weight after BC [6–9]. This fact can be related to age and nutritional status at diagnosis, therapy used during the treatment, tumour characteristics, menopausal status [10], reduced basal metabolic rate and total energy expenditure, decreased physical activity, sleep disorders, abnormal thyroid function [11] and frequent use of steroids during CT [12]. In addition, our group recently found that women with BC on endocrine therapy with Tamoxifen are mostly overweighed and obese, most evidently in women who received CT, and who were at the beginning of the treatment [13].

In this context, the guidance of proper eating habits during CT is challenging for health professionals. The side effects during the treatment may cause reduction of pleasure, and patients may also opt to eat less healthy foods and to use them as reward for the treatment [14]. Based on the above considerations, we suggest that CT has a significant impact on perceptions related to food intake, and the most significant changes occur at the end of treatment (because of the cumulative treatment effect) [15,16]. The aim of this study was to investigate the impact of CT on perceptions related to food intake at three time points over the course of CT.

## Materials and methods

### Ethical aspects

The Human Research Ethics Committee approved this study (protocol number 721.977/14), and all participants signed a free and informed consent form.

### Study design

This prospective study was conducted in a Brazilian university clinical hospital including three sequential assessments with BC patients during first-line CT.

### Eligibility criteria

In this study, we included women aged 18 years or older, diagnosed with BC, who were in the first cycle of first-line CT and who had the physical, verbal and cognitive ability needed to respond to the tools necessary for data collection.

### Database

Data collection was performed from August 2014 to October 2015. The volunteers were selected while awaiting medical consultation in the waiting room of the cancer center of this hospital.

Clinical, hormonal and therapeutic characteristics were obtained at T0 (day of the first cycle of CT infusion). Data about perceptions related to food intake and anthropometric parameters were collected at three periods during CT: T0, T1 (day of the intermediate cycle of CT infusion) and T2 (day of the last cycle of CT infusion). Side effects were evaluated in the follow-up consultations, 21 days after T0, T1 or T2 and before CT infusion. The intermediate cycle varied depending on the regimen used. When we used FAC (5-fluorouracil, adriamycin and cyclophosphamide) and CMF (cyclophosphamide, methotrexate and 5-fluorouracil), the intermediate cycle was the third. When we used AC➔Docetaxel (adriamycin and cyclophosphamide followed by docetaxel) and AC➔Paclitaxel (adriamycin and cyclophosphamide followed by paclitaxel) regimens, the intermediate cycle was the fourth.

#### Anthropometric parameters

Anthropometric parameters were obtained in the three periods of treatment (T0, T1 and T2) before CT infusion. Body weights and heights were measured using a calibrated automatic height and weight scale (Model P-150 C, Lider Balanças®, Brazil). For the measurement of waist circumference (WC), a flexible and inelastic tape was used, following the protocol recommended by the World Health Organization (WHO) [17]. Body mass index (BMI) was calculated as weight divided by height squared (kg/m^2^).

#### Perceptions evaluation related to food intake

A visual analogue scale (VAS) (0 to 10 cm) for hunger, enjoyment of eating foods and appetite for various food categories [18–20] was applied in T0, T1 and T2 before CT infusion (totalling three evaluations).

To assess hunger, patients were asked the question, “How hungry were you before the meal?”, and the researchers used a VAS of 10 cm (with “not at all” on the left and “very hungry” on the right). To assess enjoyment of eating foods, the patients were asked the question, “How much did you enjoy your food?” in a similar scale of 10 cm (with “not at all” on the left and “very” on the right). The appetite for certain food groups was verified by VAS of 10 cm (with “no desire” on the left and “a lot of will” on the right). The categories of foods assessed were starchy foods; legumes; vegetables; meat, poultry, fish and eggs; soups, broths and scalded; fruits; fruit juices; milk; dairy products; salty foods; sweet foods; acid foods; bitter foods and spicy foods. The patients were instructed to answer this question based on their appetite before a meal without worrying about nutritional issues.

In order to adapt this form to the population of interest and enable their use by the performing team, this instrument was applied in a pilot study with 15 women diagnosed with BC receiving CT in the same institution.

The hospital offered meals to patients while waiting for medical consultation and CT infusion. The researchers monitored the participant permanence in the institution, and the instrument related to food intake was applied immediately after the meal. If the patient did not eat any food while waiting in the hospital, the reason for non-intake was questioned and recorded. The interviewer questioned the patients that ate in the hospital about their reasons for eating, determinants of the food choices and how they felt after the meal.

#### Side effects related to chemotherapy

In the follow-up consultations (Δt = 21) after T0, T1 and T2, participants were questioned about the presence of secondary events, by any intensity graduations, during CT in the last seven days (i.e., taste changes, dry mouth, nausea, vomiting, constipation, diarrhoea, reduced appetite, pain and fatigue). To assess the intensity of symptoms, all items had response categories with four levels from “not at all” to “very much”. Therefore, the higher the score, the more severe the symptom was for the patient (range 1–4).

### Statistics

All the eligible women during the time of the study were invited to participate. The sample size required for this study was determined using G*Power software, version 3.1 [21]. The sample size calculations were based on an F test, ANOVA repeated measures with the effect size of F equaling 0.25, an alpha level of 0.05, 95% power, one group of individuals and three measurements. A total sample of 43 women was required at final follow-up, having been the result of the calculation that required the larger minimum sample. Considering a 20% adjustment for possible losses, a minimum of 52 women was needed at baseline (T0).

Statistical analyses were carried out using GraphPad Prism® software, version 5.0. Data distribution was determined by the Kolmogorov-Smirnov test. To check the difference between the appetite scores for food groups between T0, T1 and T2, we used the Kruskal-Wallis test with post-hoc Dunn test. Friedman and post-hoc Dunn tests assessed the variation between the three hunger scores, meal enjoyment, intensity of side effects related to CT, overall appetite and anthropometric parameters. The difference between the frequencies of the determinants of food choices, the reasons to eat and not eat, the state after meal and the side effects related to food intake were verified by Cochran's Q test and McNemar test. Confidence intervals (CI) of 0.95 and p values < 0.05 were considered statistically significant.

## Results

**Fig 1:**
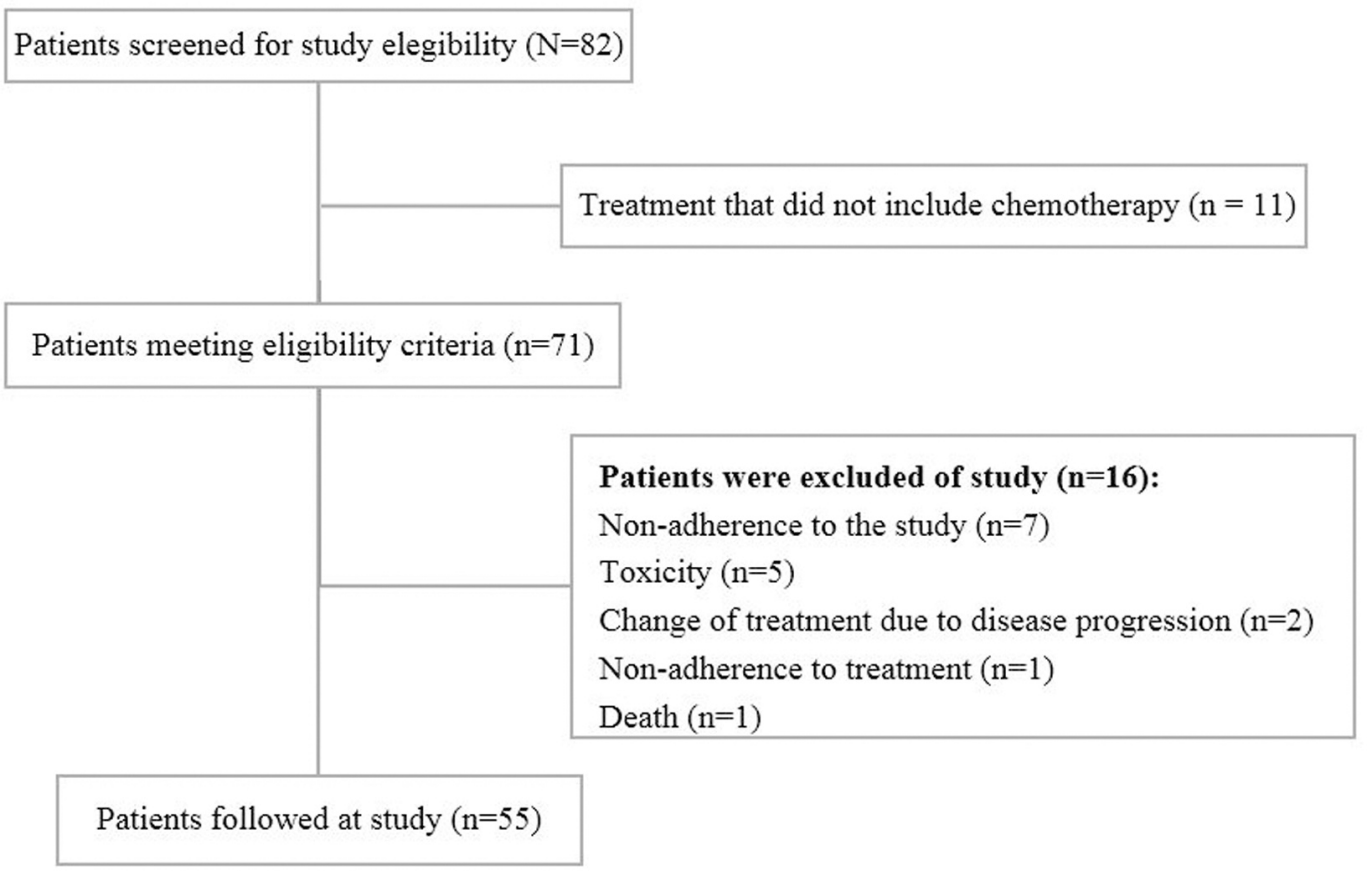
Diagram reporting the numbers of individuals at each stage of the study. Diagram reporting numbers of women with breast cancer screened, approached and recruited (n = 55).

This study included 55 women with a mean age of 51.5 ± 10.1 years. Fig 1 reports the numbers of women screened, approached and recruited to study. The results are reported according to the guidelines established by Strengthening the Reporting of Observational Studies in Epidemiology (STROBE).

Concerning clinical and hormonal characteristics, 61.8% (n = 34) were postmenopausal women; 96.4% (n = 53) had invasive ductal carcinoma; 47.3% (n = 26) were at clinical stage II and 58.2% (n = 32) had moderately differentiated tumours. Regarding the molecular subtype, the greater percentage of patients (41.8%, n = 23) was classified as luminal B. Among the patients who had undergone surgical procedures (n = 32), 75% (n = 24) underwent breast-conserving surgery. The percentage of patients that underwent adjuvant CT was 58.2% (n = 32), and the majority of patients (60%, n = 33) were treated with the AC➔Docetaxel regimen (Table 1).

**Table 1:** Clinical, anthropometric and therapeutic characteristics of women with breast cancer in chemotherapy (n = 55).

Variable	Mean ± SD or n (%)
**Age (years)**	51.5 ± 10.1
**Weight (kg)**	70.8 ± 16.4
**Height (m)**	1.5 ± 0.1
**BMI (kg/m^2^)**	28.3 ± 6.4
**WC (cm)**	90.7 ± 15.7
**Tumour Subtype**	
Invasive ductal carcinoma	53 (96.4)
Invasive lobular carcinoma	2 (3.6)
**Clinical Stage**	
I	11 (20.0)
II	26 (47.3)
III	14 (25.5)
IV	1 (1.8)
NR	3 (5.5)
**Menopausal Status**	
Premenopausal	21 (38.2)
Postmenopausal	34 (61.8)
**Chemotherapy**	
Adjuvant	32 (58.2)
Neoadjuvant	23 (41.8)
**Chemotherapy Regimen**	
AC➔ Docetaxel	33 (60.0)
AC➔Paclitaxel	8 (14.6)
FAC	9 (16.4)
CMF	5 (9.1)

SD, standard deviation; BMI, body mass index; WC, waist circumference; NR, not reported; AC➔Docetaxel, adriamycin, cyclophosphamide and docetaxel; AC➔Paclitaxel, adriamycin, cyclophosphamide and paclitaxel; FAC, 5-fluorouracil, adriamycin and cyclophosphamide; CMF, cyclophosphamide, methotrexate and 5-fluorouracil.

Concerning the anthropometric parameters, most women were overweight (T0 = 56.36%, and T1 = T2 = 54.54%), and there was a slight increase in weight between periods. However, the mean BMI corresponded to an overweight status and was higher in T1 (67.2 kg/m^2^; range 58.6–83.9 kg/m^2^) and T2 (66.4 kg/m^2^; range 58.5–83.1 kg/m^2^) than in T0 (66.1 kg/m^2^; range 58.2–84.1 kg/m^2^; p < 0.01). In addition, there was an increase in WC during the course of treatment (p = 0.03), but this difference was not detected in post-hoc tests (Table 2).

**Table 2:** Anthropometric parameters of women with breast cancer (BC) in the beginning (T0), intermediate (T1) and end (T2) of chemotherapy (CT) (n = 55).

Variable	T0	T1	T2	p-value
	Median (p25–p75) Mean ± SD or n (%)	Median (p25–p75) Mean ± SD or n (%)	Median (p25–p75) Mean ± SD or n (%)	
Weight (kg)	66.1 (58.5–84.1)^a^ 70.85 ± 16.41	67.2 (58.6–83.9)^b^ 71.38 ± 16.58	66.4 (58.5–83.1)^b^ 71.76 ± 16.79	**0.008**
BMI (kg/m^2^)	26.4 (23.5–33.7)^a^ 28.37 ± 6.42	26.3 (23.8–33.6)^b^ 28.57 ± 6.50	26.5 (23.9–33.3)^b^ 28.72 ± 6.52	**0.009**
WC (cm)	86.5 (78.5–105.0)^a^ 90.75 ± 15.66	88.0 (79.0–103.0)^a^ 91.23 ± 15.50	87.0 (80.0–103.5)^a^ 91.09 ± 15.25	**0.030**

SD, standard deviation; BMI, body mass index; WC, waist circumference; T0, day of the first cycle of CT infusion; T1, day of the intermediate cycle of CT infusion and T2, day of the last cycle of CT infusion. Medians horizontally followed by different letters differ statistically, as post-hoc test at 5% probability (Friedman test + Dunn’s post-hoc).

Considering whether there was a difference in the frequency of side effects related to food intake between the three periods, it was found that taste changes (p = 0.044) were more prevalent in the intermediate cycle of CT (T1), and the participants presented more nausea (p = 0.018) at T1 and T2. Furthermore, the intensity of nausea was higher in T2 than in T0 (p = 0.01). Although prevalence rates had not changed during treatment, dry mouth (p = 0.66), pain (p = 0.32), reduced appetite (p = 0.12) and constipation (p = 0.68) were symptoms frequently reported (Table 3).

**Table 3:** Relative frequencies and intensity of side effects related to food intake of women with breast cancer (BC) in the beginning (T0), intermediate (T1) and end (T2) of chemotherapy (CT) (n = 55).

Variable	T0	T1	T2	p-value
	Mean ± SD or n (%)	Mean ± SD or n (%)	Mean ± SD or n (%)	
Taste changes	2.00 ± 1.27 24 (43.64)^a^	2.21 ± 1.18 35 (63.64)^b^	1.92 ± 1.15 26 (47.27)^a^	0.0607 **0.044**†
Dry mouth	2.30 ± 1.23 38 (69.09)	2.52 ± 1.27 38 (69.09)	2.25 ± 1.23 34 (61.82)	0.2141 0.657†
Nausea	1.16 ± 0.53^a^ 6 ^a^ (10.91)	1.40 ± 0.83^a,b^ 13 ^b^ (23.64)	1.60 ± 1.01^b^ 18 ^b^ (32.73)	0.0119 **0.018**†
Vomiting	1.10 ± 0.49 3 (5.45)	1.12 ± 0.51 4 (7.27)	1.16 ± 0.60 5 (9.09)	0.7548 0.741†
Reduced appetite	1.63 ± 1.12 16 (29.09)	1.56 ± 1.01 16 (29.09)	1.87 ± 1.18 24 (43.64)	0.2310 0.118†
Pain	2.05 ± 1.09 32 (58.18)	2.07 ± 1.15 32 (58.18)	2.52 ± 1.28 38 (69.09)	0.0645 0.325†
Constipation	1.63 ± 1.02 19 (34.55)	1.78 ± 1.13 21 (38.18)	1.63 ± 1.07 17 (30.90)	0.6233 0.679†
Diarrhoea	1.18 ± 0.54 7 (12.74)	1.18 ± 0.58 6 (10.91)	1.27 ± 0.78 7 (12.73)	0.8825 0.946†
Fatigue	1.87 ± 1.15 25 (45.45)	1.87 ± 1.05 27 (49.09)	2.16 ± 1.18 33 (60.0)	0.1193 0.22†

SD, standard deviation; T0, day of the first cycle of CT infusion; T1, day of the intermediate cycle of CT infusion and T2, day of the last cycle of CT infusion. The side effects related to food intake were evaluated in the follow-up consultations (Δt = 21) after T0, T1 and T2. Frequencies and means horizontally followed by different letters differ statistically, as post-hoc test at 5% probability (Friedman test + Dunn’s post-hoc).

†Cochran's Q test + McNemar’s post- hoc. To assess the intensity of symptoms, the items had response categories with four levels from “not at all” to “very much” (range 1–4).

Of the 55 patients evaluated, only 23 (41.8%) ate meals in the three periods. The evaluation of the perceptions related to food intake (at T0, T1 and T2) did not show statistically significant differences between the frequencies of determinants of food choices, the reasons to eat and not eat and the state after the meal (Table 3). The taste and availability of food were the main determinants of food choices at T0, T1 and T2. Among the reasons to eat in the hospital, patients reported the habit of eating at a certain time and hunger, and the main reason for not eating was nausea. Most patients (T0 = 78.26%, T1 = 73.91% and T2 = 56.52%) were satiated after meals, which were identified by a hunger scale corresponding from average to moderate hunger with no statistically significant difference between the times (p = 0.113) (Table 4).

**Table 4:** Frequencies of determinants of food choice, reasons to eat or not to eat, state after the meal and median (p25–p75) of hunger and food satisfaction scales of women with breast cancer (BC) in chemotherapy (CT) (n = 23).

Variable	T0		T1		T2		p-value
	n	% or Median (p25–p75)	n	% or Median (p25–p75)	n	% or Median (p25 –p75)	
**Determinants of food choice**							
Appetite	4	17.39	2	08.69	0	00.00	0.055
Food habit	3	13.04	5	21.73	1	04.34	0.180
Available time	4	17.39	4	17.39	2	08.69	0.641
Available Food	7	30.43	11	47.82	10	43.47	0.522
Taste	9	39.13	7	30.43	12	52.17	0.368
**Reasons to eat**							
Always eat at this time	8	34.78	4	17.39	7	30.43	0.307
Hunger	6	26.08	10	43.47	8	34.78	0.397
Follow friends/Family	1	04.34	2	08.69	0	00.00	0.368
Friends/family request	1	04.34	0	00.00	0	00.00	0.368
Follow medical/nutritional guidance	3	13.04	5	21.73	5	21.73	0.607
Because they offered	4	17.39	3	13.04	5	21.73	0.741
**Reasons not to eat**							
Do not eat at this time	0	00.00	0	00.00	1	04.34	0.368
Reduced apetite	1	04.34	1	04.34	0	00.00	0.607
I had nothing to eat	1	04.34	0	00.00	0	00.00	0.368
I didn’t like what I had to eat	1	04.34	0	00.00	0	00.00	0.368
Nausea	0	00.00	2	08.69	1	04.34	0.223
The smell bothers me	0	00.00	1	04.34	0	00.00	0.368
Severe pain	1	04.34	0	00.00	0	00.00	0.368
**State after the meal**							
Still hungry	3	13.04	2	08.69	5	21.73	0.368
Satisfied	18	78.26	17	73.91	13	56.52	0.174
Stuffed	2	08.69	1	04.34	1	04.34	0.779
Nauseated	0	00.00	2	08.69	4	17.39	0.091
With “burning” in the stomach	1	04.34	1	04.34	0	00.00	0.607
Hunger	23	5.0 (2.0–5.0)	23	5.0 (3.0–8.0)	23	5.0 (2.0–6.0)	0.113†
Meal enjoyment	23	9.0^a^ (7.0–10.0)	23	6.0^b^ (5.0–10.0)	23	9.0^a^ (8.0–10.0)	**0.021**†

T0, day of the first cycle of CT infusion; T1, day of the intermediate cycle of CT infusion and T2, day of the last cycle of CT infusion. Frequencies horizontally followed by different letters differ statistically, as post-hoc test at 5% probability (Cochran's Q test + McNemar’s post hoc).

† Friedman test + Dunn’s post-hoc; n = 23 because 32 women do not eat during the hospital stay, making it impracticable compare the individual with him/herself. Subjects were allowed to choose more than one option for each question. To evaluate hunger and meal enjoyment, a visual analogue scale (VAS) (0 to 10 cm) was used.

The appetite for salty foods was greater in T1 and T2 than in T0 (p < 0.01), while spicy foods were best assessed in T1 (p = 0.03). Fruits and fruit juices had higher scores, but they did not differ between the three periods. The overall appetite increased during the course of CT (T0–T2; p = 0.0028; Table 5).

**Table 5:** Scores of appetite for food groups of women with breast cancer (BC) in the beginning (T0), intermediate (T1) and end (T2) of chemotherapy (n = 55).

Food categories	T0	T1	T2	p-value
	Median (p25–p75)	Median (p25–p75)	Median (p25–p75)	
Starchy foods (breads, cookies, potato, rice)	5.0 (2.0–8.0)	6.0 (1.5–9.25)	7.0 (4.0–10.0)	0.211
Legumes (chick peas, beans, peas, lentils)	0.0 (0.0–5.0)	0.0 (0.0–6.0)	0.0 (0.0–8.0)	0.970
Vegetables (pumpkin, chayote, tomato, carrot)	6.0 (0.0–8.0)	0.0 (0.0–9.25)	0.0 (0.0–8.25)	0.553
Meat, poultry, fish and eggs	0.0 (0.0–8.0)	0.0 (0.0–8.0)	0.0 (0.0–7.25)	0.749
Soups, broths and scalded	0.0 (0.0–7.0)	0.0 (0.0–8.25)	0.0 (0.0–9.0)	0.875
Fruits	7.0 (4.0–10.0)	8.0 (5.75–10.0)	8.0 (5.0–10.0)	0.525
Fruit juices	8.0 (5.0–10.0)	8.0 (3.75–10.0)	8.5 (6.5–10.0)	0.587
Milk	0.0 (0.0–8.0)	3.5 (0.0–8.0)	1.5 (0.0–8.5)	0.753
Dairy products (yogurt, cheese)	5.0 (0.0–8.0)	6.0 (0.0–9.0)	7.0 (0.0–10.0)	0.517
Salty foods (snacks, nuts, olives)	5.0ª (0.0–9.0)	8.0ᵇ (4.75–10.0)	8.0ᵇ (6.0–10.0)	**0.004**
Sweet foods (sweet guava, ice cream, dulce de leche)	3.0 (0.0–9.0)	5.0 (0.0–8.0)	5.5 (0.0–8.0)	0.541
Acid foods (lemon popsicle, acerola juice)	3.0 (0.0–6.0)	5.0 (0.0–8.0)	5.0 (0.0–8.0)	0.262
Bitter foods (jilo, chicory, eggplant)	4.0 (0.0–8.0)	6.5 (0.0–10.0)	6.5 (0.0–9.25)	0.212
Spicy foods (ketchup, pepper)	0.0ª (0.0–4.0)	4.0ᵇ (0.0–8.25)	2.5ª^,b^ (0.0–7.0)	**0.036**
*Overall appetite*	3.5ª (0.0–5.25)	5.0ª^,b^ (0.0–6.7)	5.25ᵇ (0.0–7.25)	**0.0028**†

T0, day of the first cycle of CT infusion; T1, day of the intermediate cycle of CT infusion and T2, day of the last cycle of CT infusion. Medians horizontally followed by different letters differ statistically, as post-hoc test at 5% probability (Kruskal-Wallis test + Dunn’s post-hoc).

†Friedman test + Dunn’s post-hoc. For the measurement of appetite, a visual analogue scale (VAS) (0 to 10 cm) was used.

## Discussion

The results of this prospective study, which investigated women with BC receiving CT, support the hypothesis that polychemotherapy alters food hedonism by increasing the overall appetite and appetite for salty and spicy foods. In addition, the treatment has a negative impact on meal enjoyment and on the manifestation of side effects, such as taste changes and nausea. Despite the negative impact of CT, meal enjoyment, weight, BMI and WC have increased. Therefore, this study provides relevant knowledge to help understand the impact of CT on perceptions related to food intake and consequent changes in meal appreciation and nutritional status among women with BC.

Taste changes are important adverse events during CT and can affect 38% to 84% of cancer patients [16]. In the present study, the prevalence of taste changes was higher at T1 than at T0 and T2 (p = 0.04). A higher prevalence of taste changes was expected at T2 (due to the cumulative effect of CT), but, at the end of treatment, taste was recovered (which may be due to the individual's ability to adapt) [22]. From T0 to T1, we found a reduction in the scores for meal enjoyment (p = 0.021). Reinforcing our findings, Bernhardson et al. [23] mentioned that modifications of smell/taste cause feelings of disappointment, irritation, boredom, sadness and melancholy; and, because of that, patients reported a reduced sense of satisfaction or comfort with respect to food.

Despite the side effects of CT, the hunger scale showed moderate levels in the three periods (T0, T1 and T2; median = 5). In spite of the fact that hunger had not been modified, the overall appetite increased. Although these terms are often used interchangeably—hunger is the lack of fullness, while appetite is the desire to eat [24]. According to Drapeau et al. [25], the desire to eat, prospective food consumption and hunger are related in a positive form with the total energy intake, while the feeling of fullness is correlated in a negative way with this same variable. Also, the administration of CT is associated with anxiety and depression [26], which can cause reduced appetite [27]. High anxiety levels could reduce the motivation for food intake and the amount of pleasure during a meal [28]. In the last cycle of CT, women may have felt relieved and less anxious/depressive, which may explain the increase in overall appetite in T2. Another hypothesis is that appetite may have increased due to the use of corticosteroids [29] during therapy.

Related to the appetite for some food groups, salty foods obtained higher scores in T1 and T2 (T0 = 5, T1 = 8 and T2 = 8). It seems that the appetite for salty foods is more influenced by environmental factors than by individual genetic backgrounds [30], corroborating our findings, since exposure to CT agents has led to a change in appetite for this taste. Boltong et al. [31], in a study performed with 52 women with BC receiving adjuvant CT, showed a reduction in the capability to correctly identify salty flavours during treatment. In our study, the appetite for spicy foods was higher in T1 (T0 = 0, T1 = 4 and T2 = 2.5). Byrnes and Hayes [32] verified that patients prefer spicy foods and also exhibit higher searches for perceptions and greater sensitivity to rewards. Capsaicin, which is the principal pungent substance in chili peppers, evokes oral burning [33] through oral sensory stimulation [34]. In the present study, the hedonic alterations occurred at the period that taste changes were higher (T1), and the meal enjoyment had lower scores (T1), justifying the choice for more sensorial food stimuli. In a qualitative cross-sectional study, both patients and family members mentioned that an alternative to increase food flavouring was the inclusion of flavour enhancers or an increase sodium based condiments (e.g., salt, ginger, soy sauce and Worcestershire sauce) [5]. Steinbach et al. [34] also support this strategy suggesting the addition of seasoning and spices to preparations. Fruits and fruit juices had higher scores but did not differ between T0, T1 and T2 (which is consistent with the literature), since some women after the diagnosis of BC follow a healthy diet [35].

Other factors besides altered taste and appetite can influence meal enjoyment, including nausea and vomiting induced by chemotherapy (CINV). These side effects are well recognised in cancer patients and can be divided into acute symptoms (until 24 hours after CT) or late symptoms (typically between the second and the fifth day after CT) [36]. Studies indicate that the prevalence of these symptoms varies from 38% to 60% during CT [37–39]. When severe, nausea can affect food intake and patient functional capacity [2]. In the present study (after the first CT cycle), 10.91% (n = 6) of patients reported nausea, reaching 32.73% (n = 18) after the last CT cycle (p = 0.018). Castro et al. [40] investigated the prevalence of CINV in 42 women with BC in the same institution and found 52.4% of patients had late-onset nausea. The highest prevalence found by these authors is explained by CINV being evaluated up to 4 days after CT infusion, while we evaluated CINV on the infusion day of the next CT cycle (i.e., after 21 days).

The food choice is the result of a complex interaction between intra- and interpersonal factors [41]. The determinants of food choices can be classified as biological (hunger, taste and appetite), economic (cost, income and availability), physical (access, education, cooking facilities and time), social (culture, family, peers and meal patterns), and psychological (disposition, stress and blame), besides attitudes, beliefs and knowledge about food [42]. In the general population, food choice is based on taste, cost, convenience, health and variety, and the taste is perceived as a highly important factor in food choice decisions [30]. According to Poortvliet et al. [43], the participants liked some specific meal due to the use of fresh ingredients, the diversity, the flavour, the quality and the color of the repast. In this study, the main determinants of food choice were taste and food availability, confirming that taste also has great influence on the eating behaviour of women with BC. In fact, the "taste" is the sum of all the sensory stimuli that are produced during food intake [44]. Probably, due to the fact that these patients only had access to meals served by the institution or brought from home while waiting for medical consultation, food availability had an important impact on food choice. Among the reasons to eat or not to eat, we highlight individual aspects (biological determinants and attitudes about food) in that decision, since hunger and the habit of food at a certain time were the main reasons for eating and the most common reason for not eating was nausea (reinforcing the importance of this symptom).

The cytotoxicity of CT possibly reduces the numbers of taste and smell receptors, resulting in taste loss. Other possible explanations for taste loss include changes in the rate of turnover of receptor cells, changes in the structure of receptors affecting the delivery of taste and smell molecules to taste and smell receptors or abnormalities in the re-establishment of synaptic connections at the end of treatment [45]. Studies suggest that treatment for malignancies may have an influence on food preferences through the development of food aversions [46]. There are reports that the likelihood of an individual selecting a food for a second time is related to their prior experiences [47]. This may be relevant to the development of food aversions in the setting of CT, as taste and smell alterations during the disease and subsequent treatments coupled with side effects may have resulted in negative experiences during feeding [48,49]. In addition, the taste dysfunction has also been related to obesity [50].

Related to anthropometric parameters, most women were overweight (T0 = 56.36% and T1 = T2 = 54.54%), and there was an increase in body weight (p = 0.008), BMI (p = 0.009) and WC (p = 0.03) during the course of CT. Although these alterations were not clinically significant, it shows a tendency for weight gain and an increase in visceral adiposity after the diagnosis and treatment of BC. The increase in body weight in women after the diagnosis of BC is common, affecting 50% to 95% of them [51] and is associated with a higher risk of BC recurrence and death [8,52]. A retrospective cohort study that included 271 participants with BC showed that in the first year of treatment there was a weight gain of 2 kg. Literature reported that not only an increased BMI is associated with higher risk of BC but also a larger WC [53]. Visceral adiposity is associated with hyperinsulinemia, insulin resistance, inflammation and hormonal changes, with greater bioavailability of oestrogen and testosterone in the blood [54]. Possibly the increase in anthropometric parameters may be related to increased appetite observed in these women, but more studies should be conducted to investigate the truth of this relationship. Besides, the use of corticosteroids by these women during CT may be related to fluid retention [55] and may have interfered with the anthropometric parameters.

The study has some limitations. Immediate side effects of CT (in the first two weeks) were not assessed and a diary to be completed by the patients was initially judged not feasible in the context of our patient population. In addition, side effects were not classified according to the common toxicity criteria (CTC), which are frequently reported in the scientific literature. Conversely, we decided to evaluate side effects of CT according to patient’s observations; thus, patient reported measures were considered more adequate considering our main aims. Another possible limitation of the study was the inclusion of BC patients submitted to different regimens of CT. Since the study was not powered to compare different CT regimens, further studies comparing taste changes among different regimens are warranted.

## Conclusions

The results of this study support the hypothesis that CT changes food hedonism (increasing the overall appetite and the appetite for salty and spicy foods) as well as negatively affect meal enjoyment, BMI, WC and the manifestation of secondary events, such as nausea and taste changes. These results may help to understand the perceptions related to food intake and may serve as a basis for health professionals reviewing post-diagnosis guidelines, presenting strategies for reducing these symptoms in order to improve the nutrition of patients and individualised assistance. Overall, more longitudinal studies are required to accurately define the nature, magnitude and time course of taste, food liking and appetite changes over the treatment trajectory.

## Supporting information

S1 File The database file for this manuscript (Microsoft Excel format). (XLSX)
